# Genetic regulation and heritability of miRNA and mRNA expression link to phosphorus utilization and gut microbiome

**DOI:** 10.1098/rsob.200182

**Published:** 2021-02-17

**Authors:** Siriluck Ponsuksili, Michael Oster, Henry Reyer, Frieder Hadlich, Nares Trakooljul, Markus Rodehutscord, Amélia Camarinha-Silva, Jörn Bennewitz, Klaus Wimmers

**Affiliations:** ^1^ Leibniz Institute for Farm Animal Biology, Institute for Genome Biology, Wilhelm-Stahl-Allee 2, 18196 Dummerstorf, Germany; ^2^ Institute of Animal Science, University of Hohenheim, 70599 Stuttgart, Germany; ^3^ Faculty of Agricultural and Environmental Sciences, University Rostock, 18059 Rostock, Germany

**Keywords:** miR-eQTL, mRNA-eQTL, phosphorus, mineral homeostasis, microbiota, heritability

## Abstract

Improved utilization of phytates and mineral phosphorus (P) in monogastric animals contributes significantly to preserving the finite resource of mineral P and mitigating environmental pollution. In order to identify pathways and to prioritize candidate genes related to P utilization (PU), the genomic heritability of 77 and 80 trait-dependent expressed miRNAs and mRNAs in 482 Japanese quail were estimated and eQTL (expression quantitative trait loci) were detected. In total, 104 miR-eQTL (microRNA expression quantitative traits loci) were associated with SNP markers (false discovery rate less than 10%) including 41 eQTL of eight miRNAs. Similarly, 944 mRNA-eQTL were identified at the 5% False discovery rate threshold, with 573 being cis-eQTL of 36 mRNAs. High heritabilities of miRNA and mRNA expression coincide with highly significant eQTL. Integration of phenotypic data with transcriptome and microbiome data of the same animals revealed genetic regulated mRNA and miRNA transcripts (SMAD3, CAV1, ENNPP6, ATP2B4, miR-148a-3p, miR-146b-5p, miR-16-5p, miR-194, miR-215-5p, miR-199-3p, miR-1388a-3p) and microbes (*Candidatus *Arthromitus*, Enterococcus*) that are associated with PU. The results reveal novel insights into the role of mRNAs and miRNAs in host gut tissue functions, which are involved in PU and other related traits, in terms of the genetic regulation and inheritance of their expression and in association with microbiota components.

## Graphical abstract

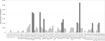

## 1. Introduction

Phosphorus (P) is an essential mineral and a limited resource. The excessive use of mineral P in agricultural production has an environmental impact. Consequently, vigilant P management is required. The bulk of P in animal feeds originates from plant seeds. However, up to 80% of plant P is in the form of phytates, which cannot be efficiently used by monogastrics due to the lack or scarcity of endogenous phytase in their digestive tract. Microbial phosphatases may contribute to the cleavage of P from phytates in the gut of monogastric animals. Gut properties, the microbiome composition and interactions between the gut tissue and microbiota play a significant role for digestive capacity and need to be understood for improvement of phosphorus utilization (PU). The most promising molecules mediating between gut microbiota and host are microRNAs (miRNAs), which are conserved between species and can regulate gene expression across species. We previously identified differentially abundant miRNAs, mRNAs and microbiota in the gut of Japanese quail representing extremes for PU [1,2].

MiRNAs are small endogenous non-coding molecules ranging from 18 to 24 nt that control target transcripts by inducing transcript cleavage, degradation, destabilization and repression of translation, thereby modulating protein levels. MiRNAs contain a 6–8 nt section known as seed sequence [3]. This sequence is important for targeting mRNA sites via base-pair complementarity, typically in the 3′-untranslated region [4,5] but also in the coding sequence [6]. Since a single miRNA can regulate the expression of several genes and a single gene can be influenced by several miRNAs, it was assumed that more than 50% of the genes are controlled by miRNA [7]. Moreover, miRNA-expression profiles have been associated with many complex traits and diseases [8–10]. Transcript abundances are endogenous traits that represent an important contribution to the expression of complex traits and that depend on genetic polymorphisms (i.e. they are heritable). Genetic variation associated with variation of expression levels can be investigated through expression-QTL (eQTL) analysis. Many eQTL mapping studies reveal genetically regulated mRNA transcripts in various species and tissues. However, miRNA-eQTL studies are still sparse [11–16]. The heritability of transcript abundances of various miRNAs and mRNAs is one criterion to prioritize candidates for further analyses [17].

Our previous study in an experimental F2 population of Japanese quail showed genetic parameters of PU, including a low heritability of 0.14 and a causal structure indicating that a high PU is associated with a low gain per feed and a high body weight gain [18]. In addition, PU was shown to have a strong genetic and phenotypic correlation with bone ash, a trait with a much higher heritability of 0.23–0.34 [19]. New molecular pathways affecting PU were identified by detecting differentially expressed miRNAs and mRNAs in the ileum of PU-divergent Japanese quail, and a biomarker panel associated with PU was provided [1,2]. Further studies looked at microbial gut profiles [2,20,21]. A microbial core community showed shifts in abundances associated with the PU. The list of miRNAs and mRNAs that showed differential expression in ileum tissue of Japanese quail with divergent PU serves as a basis to select transcripts to be used here in a study of a larger number of animals. The identification of SNPs associated with miRNA and mRNA abundance in the ileum has the potential to aid in the understanding of the role of gut cell functions in PU and related traits. We therefore estimated the heritability and examined the genetic regulation of ileum miRNA and mRNA expression and its consequences on mineral utilization including PU, calcium utilization (CaU), bone ash traits (femur ash absolute, F ash abs; femur ash relative, F ash rel; tibial ash absolute, T ash abs; tibial ash relative, T ash rel) and performance traits. At the same time, we considered the association of gut microbiota data obtained from the same animals with transcripts and phenotypes.

## 2. Results

After having identified differentially expressed genes, the aim of this study was to identify genetic variants impacting both miRNA and mRNA expression levels and to estimate respective heritabilities. The top differentially expressed transcripts including 77 miRNAs and 80 mRNAs were selected for genomic heritability and genetic regulation study in 482 Japanese quails.

### 2.1. Genomic heritability and genetic regulation of miRNA transcripts (miR-eQTL)

**Figure 1.**
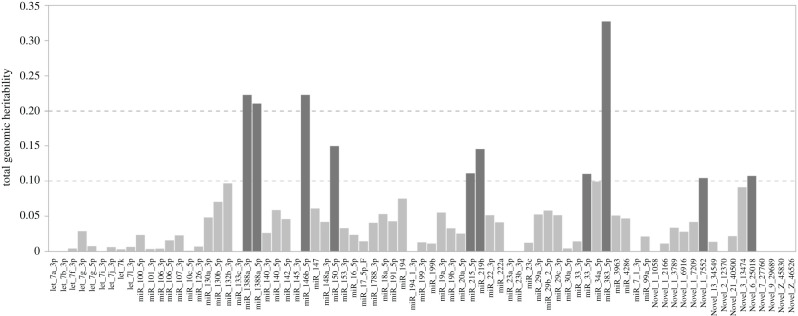
Genomic heritability of transcript abundances of 77 miRNAs observed from 482 individuals.

Estimates of genomic heritability were low and smaller than 0.1 for most miRNA-expression values, except for miR-383-5p, miR-146b, miR-1388a-3p and miR-1388-5p, which showed heritabilities of 0.21 to 0.33 (figure 1; electronic supplementary material, file S3).

**Figure 2.**
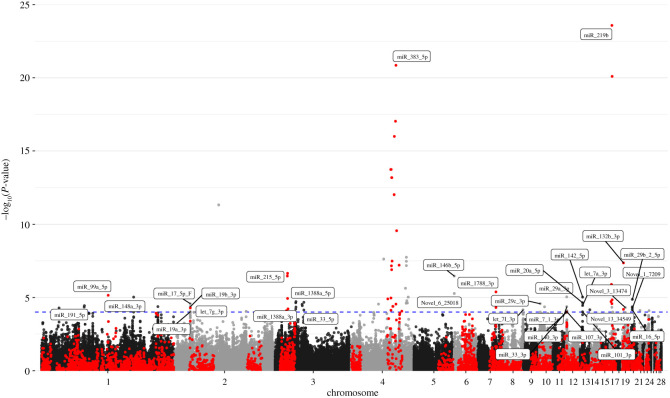
Manhattan plot (−log10[P] genome-wide association plot) of a genome-wide association study on 77 expressed miRNA in 482 quails from an F2 population. The red points indicate SNPs in cis with the respective miRNA; the black and grey points indicated markers in trans to miRNAs. The horizontal dotted line indicates the significance threshold of FDR less than 0.1 (corresponding to *p* < 9.9 × 10^−5^). The *x*-axis indicates the chromosomal position.

A total of 104 miR-eQTL corresponding to 34 miRNAs were found at *p* < 9.9 × 10^−5^, corresponding to a false discovery rate (FDR) of less than 0.1. Forty-one miR-eQTL of 8 miRNAs were identified as cis-miR-eQTL including miR-99a-5p, miR-383-5p, miR-219b, miR-215-5p, miR-1788-3p, miR-16-5p, miR-148a-3p and miR-132b-3p. Manhattan plots and the information of miR-eQTLs are shown in figure 2 and electronic supplementary material, file S4. The strongest association was found between miR-219b and SNP (id11670) on chromosome 17 position 37 kb (*p* = 2.6 × 10^−24^) and assigned to cis-miR-eQTL.

### 2.2. Genomic heritability and genetic regulation of mRNA transcripts (mRNA-eQTL)

**Figure 3.**
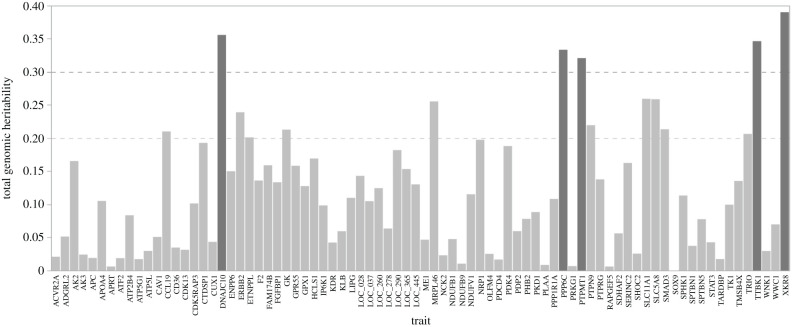
Genomic heritability of transcript abundances of 80 mRNAs observed from 482 individuals.

Five transcripts (*XKR8, DNAJC10, TTBK1, PPP6C* and *PTPMT1*) showed a genomic inheritance of more than 0.3. The modest genetic influence was observed for transcripts of *SLC11A1, SLC5A8, MRPL46, ERBB2, PTPN9, SMAD3, GK, CCL19, TRIO* and *ETNPPL* with genomic heritabilities of more than 0.2 (figure 3; electronic supplementary material, file S5).

**Figure 4.**
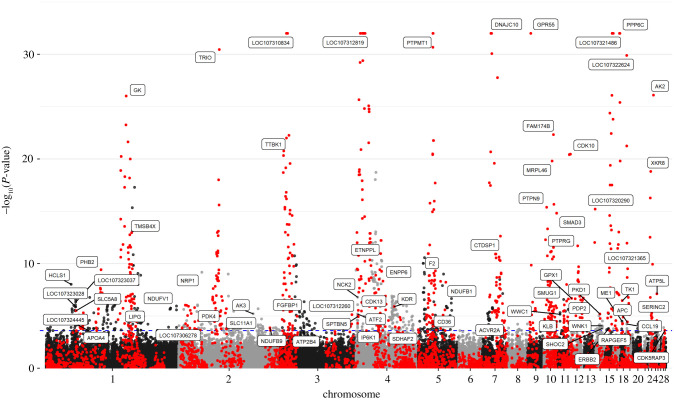
Manhattan plot (−log10[P] genome-wide association plot) of a genome-wide association study on 80 expressed mRNA in 482 quails from an F2 population. The red points indicate SNPs in cis with the respective mRNA; the black and grey points indicated markers in trans to mRNAs. The horizontal dotted line indicates the significance threshold of FDR less than 0.05 (corresponding to *p* < 1.8 × 10^−4^). The *x*-axis indicates the chromosomal positions.

A total of 944 mRNA-eQTL covering 62 mRNAs were found at 5% FDR (figure 4; electronic supplementary material, file S6). The top ten significant eQTL belong to *DNAJC10, GPR55, PPP6C, PTPMT1, TRIO, AK2, GK, FAM174B, MRPL46* and *XKR8*. 573 mRNA-eQTL were identified as cis-eQTL and correspond to 36 mRNA. Almost all of the highly significant mRNA-eQTL was assigned as cis-mRNA-eQTL. All transcripts with a genomic inheritance of more than 0.3 and most transcripts with a genomic inheritance of more than 0.2 coincided with the cis-e-QTL.

### 2.3. Common SNPs associated with mRNA and miRNA transcript abundances

Of those SNPs that showed significant association with mRNA or miRNA abundances there were 34 that were simultaneously associated with mRNA and miRNA expression; half of them were cis-eQTL (electronic supplementary material, file S7). For example, SNP id08161 was associated with the expression of miR-215-5p, and mRNAs *TTBK1* and *LOC107310834* were located in the same position. Similarly, SNPs associated with miR-1788-3p or miR-219b were also associated with *CTDSP1* or *PPP6C*, respectively. In other case, SNPs associated with miR-383-5p in cis were also associated with transcript abundances of *ETNPPL, KDR* and *LOC107312819* located on different chromosomes.

### 2.4. Trait-correlated expression

**Figure 5.**
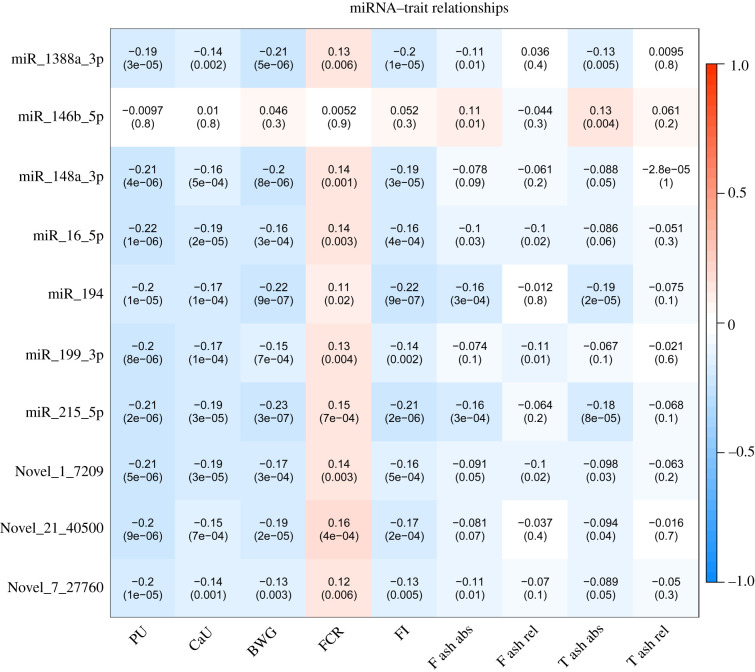
Pearson correlation coefficients and *p*-values between residuals of miRNA expression (ct value) and phenotypes (phosphorus utilization (PU), calcium utilization (CaU)), bone ash traits (femur ash absolute (F ash abs), femur ash relative (F ash rel), tibial ash absolute (T ash abs), tibial ash relative (T ash rel)) and body weight gain (BWG), feed conversion rate (FCR) and feed intake (FI)) after correction of all covariances (method). The colour bar indicates positive correlation (red) and negative correlation (blue).

**Figure 6.**
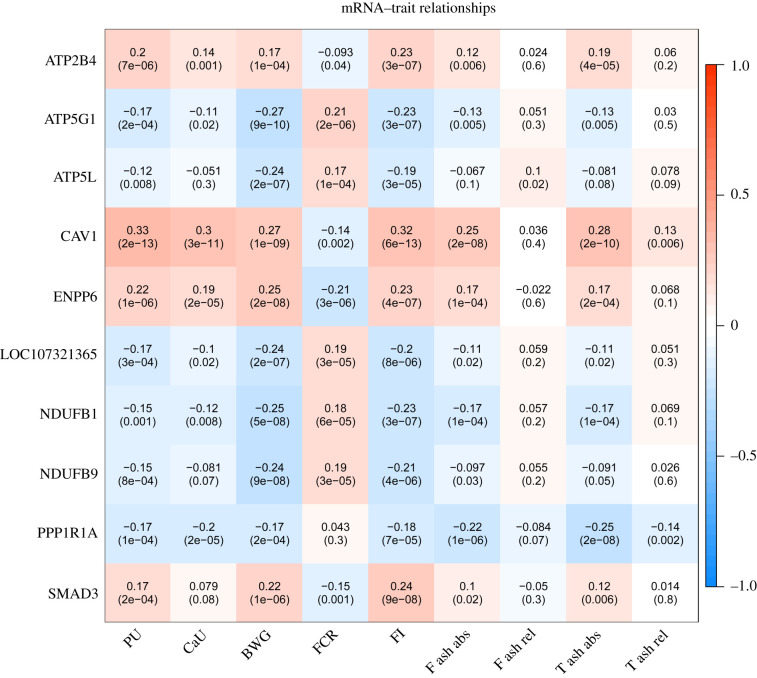
Pearson correlation coefficients and *p*-values between residuals of mRNAs expression (ct value) and phenotypes (phosphorus utilization (PU), calcium utilization (CaU)), bone ash traits (femur ash absolute (F ash abs), femur ash relative (F ash rel), tibial ash absolute (T ash abs), tibial ash relative (T ash rel)) and body weight gain (BWG), feed conversion rate (FCR) and feed intake (FI)) after correction of all covariances (method). The colour bar indicates positive correlation (red) and negative correlation (blue).

**Figure 7.**
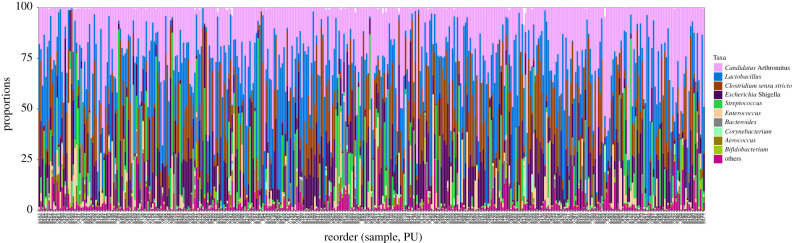
Top 10 core gut microbiota. Distribution of genera obtained from 482 Japanese quail (*Coturnix japonica*). *x*-axis indicates the normalized operational taxonomic units (OTU) using DESeq2. Each point represents a normalized OTU.

**Figure 8.**
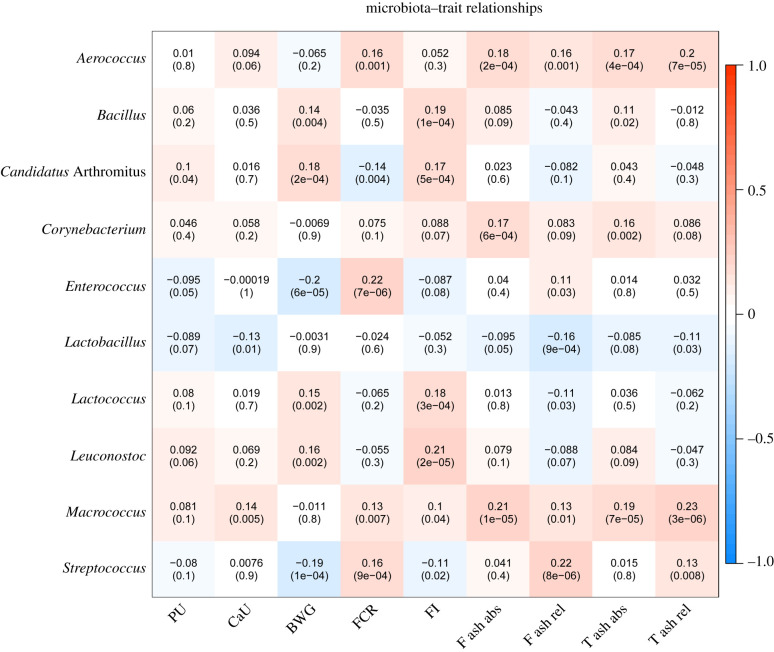
Pearson correlation coefficients and *p*-value between residuals of microbiota and phenotype (phosphorus utilization (PU), calcium utilization (CaU)), bone ash traits (femur ash absolute (F ash abs), femur ash relative (F ash rel), tibial ash absolute (T ash abs), tibial ash relative (T ash rel)) and body weight gain (BWG), feed conversion rate (FCR) and feed intake (FI)) after correction of all covariances (method). The colour bar indicates positive correlation (red) and negative correlation (blue).

**Figure 9.**
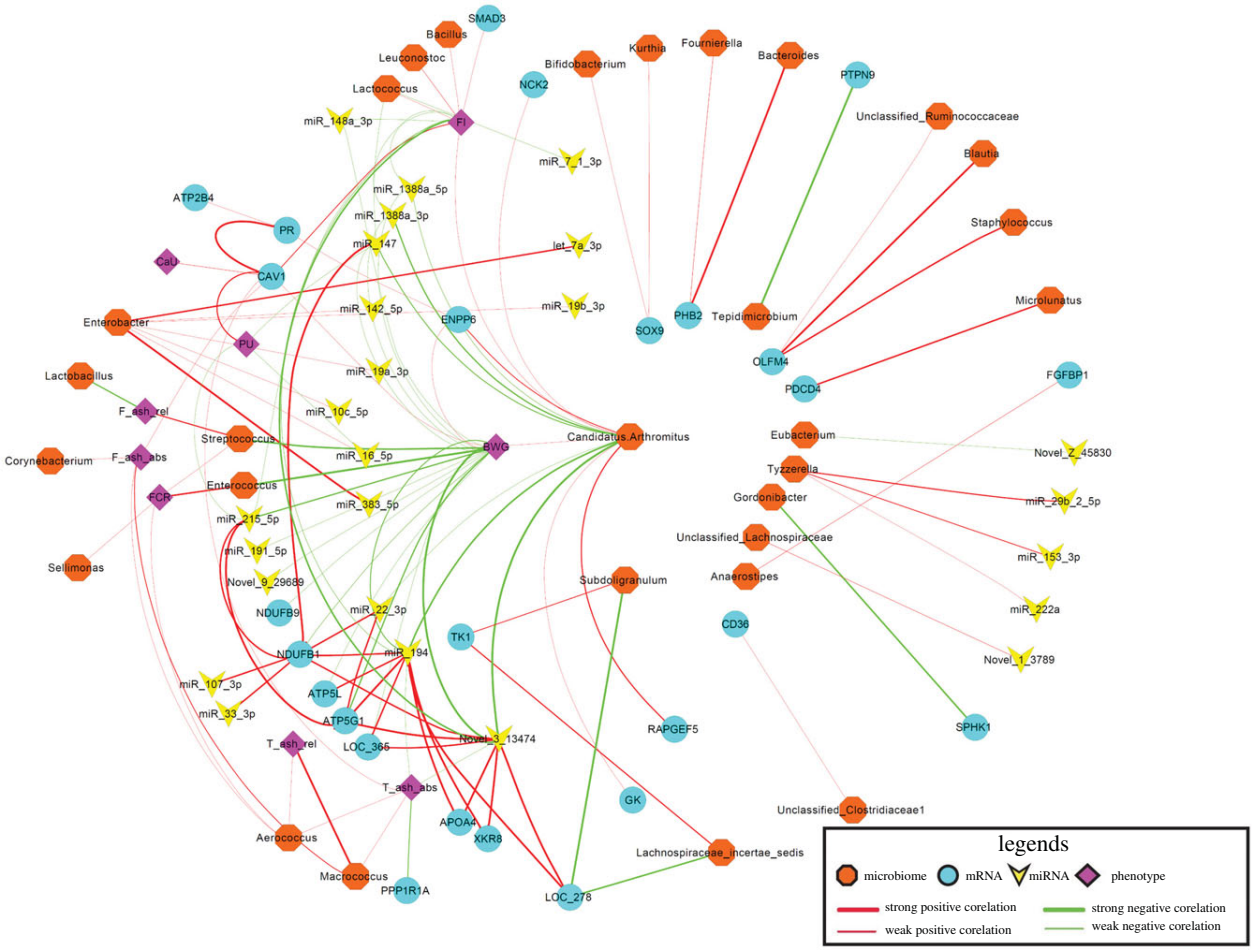
Network of top 20 links between miRNA, mRNA, microbiota and phenotypes including phosphorus utilization (PU), calcium utilization (CaU), bone ash traits (femur ash absolute (F ash abs), femur ash relative (F ash rel), tibial ash absolute (T ash abs), tibial ash relative (T ash rel)) and body weight gain (BWG) and feed conversion rate (FCR) and feed intake (FI) traits based on Pearson correlation coefficients.

Using residuals derived from models considering fixed and random effects, 66 miRNA sequence species correlated with traits related to PU (electronic supplementary material, file S8). The top traits correlated with miRNAs (miR-215-5p, miR-194, Novel-3-13474, miR-16-5p, Novel-1-7209, Novel-7-27760, miR-1388a-3p, miR-146b-5p, miR-199-3p and miR-148a-3p) are shown in figure 5. Most of these were negatively correlated with Ct value (positive with expression levels). MiR-16-5p was found highest correlated with PU (*r* = −0.22, FDR = 1.2 × 10^−4^). Likewise, expression levels of 44 mRNAs correlated with traits including *CAV1, ATP5G1, ENPP6, PPP1R1A, NDUFB1, NDUFB9, SMAD3, LOC107321365, ATP5 L* and *ATP2B4* that showed highest correlations (electronic supplementary material, file S9; figure 6). Also between the trait-correlated miRNAs and mRNAs there was negative correlation, for example for the pairs of *CAV1* and miR-1388a-3p (*r* = −0.25, *p* = 3.07 × 10^−8^), or *ADGRL2* and miR-148a-3p (*r* = −0.27, *p* = 1.46 × 10^−9^). The taxonomic characterization of the microbiota composition, including the top ten genera obtained from 411 individual Japanese quail samples that overlap with animals used in this study, is shown in figure 7. No correlation between transcripts and microbiota reached the threshold of FDR 5%. However, *Candidatus *Arthromitus** negatively correlated with miR-194, miR-1388a-3p, miR-147 and Novel-3-13474, and also positively correlated with *RAPGEF5, ENPP6* and *FGFBP1*, all of which are transcripts linking to PU. *Enterococcus* showed the strongest connection to miR-34a-5p and IP6K1 as well as FCR. In total, 14 microbial taxa correlated with one of the PU related traits at FDR less than 5% (electronic supplementary material, file S10; figure 8). *Macrococcus* was highly positive correlated with T ash rel, F ash abs and T ash abs (*r* = 0.19–0.23, FDR less than 1%). *Enterococcus, Streptococcus* and *Candidatus *Arthromitus** were strongly linked to BWG at FDR less than 5%. The network showed the top 20 links between miRNA, mRNA, microbiota and phenotype (figure 9). The figure illustrates a strong negative association between *Candidatus *Arthromitus** and some miRNAs, including miR-1388a-5p, miR-1388a-3p, miR-147, miR-194 and Novel-3-13474, and a positive correlation to *ENPP6* and *RAPGEF5*.

## 3. Discussion

Extremes for PU traits out of an F2 population of Japanese quail from our previous study reveal molecular interactions occurring in the gut of quail and provided a number of biomarker panels associated with the PU [2]. These biomarkers, which include differentially expressed mRNAs, miRNAs and their targets, are not only linked to PU traits, but also to microbiota enriched in extremes for PU traits. In addition, the mRNA, miRNAs and its targets were enriched in functional pathways involved in energy metabolism, cell proliferation, phosphate or bone metabolism, as well as immune pathways [1,2]. Here we further focused on selected mRNA and miRNA transcripts by estimating heritabilities of expression levels and detecting eQTLs. The molecular traits affect the phenotype at the organismal level including traits related to mineral homeostasis such as PU, CaU and bone ash traits or performance traits such as BWG, FCR and FI. In fact, a previous study showed a strong genetic and phenotypic correlation between PU and bone ash traits which are alternative traits reflecting PU [22]. In general, a higher number of eQTL were identified for mRNA than for miRNA. Moreover, the average genomic heritability estimates were slightly higher for mRNA compared to miRNA. In addition, eQTLs of miRNAs or mRNAs in cis dominated compared to trans eQTLs in terms of number and significance, which is consistent with other studies [16,23]. Transcript abundances of a number of miRNAs and mRNAs proved to be heritable molecular features. The majority of these also had significant eQTL which is in line with a previous study [22]. For example, the expression of miR-383-5p was highly heritable and a cis-miR-eQTL existed. We found a majority of common or shared eQTLs affecting both miRNA and mRNA expression, which was also in line with previously reported results [24]. However, this previous study found that the majority of shared eQTLs affected miRNA and host expression differently [24].

In addition, a number of miRNAs and mRNAs, whose expression correlates with characteristics of the P metabolism, whose transcript abundances are heritable, and for which eQTL have thus been identified, also show functional connections with metabolic pathways of P homoeostasis and/or with the microbiota of relevance for PU (figure 9).

These interrelationships complement results from GWAS and facilitate their interpretation. Remarkably, most of miRNA and mRNA that we previously identified as differentially expressed between Japanese quail divergent for PU were confirmed in this study using a large number of animals representing the whole phenotypic distribution. In addition, the study provides a narrowed list of candidate transcripts mediating interaction between host gut microbiomes and PU characteristics. As shown in our previous study with the same F2 population of Japanese quail, intestinal microbiota are related to the above-mentioned phenotypes [2,20] and specific microbiota have shifted abundance in parallel with PU. This includes *SMAD3, CAV1, ENNPP6, ATP2B4*, miR-148a-3p, miR-146b-5p, miR-16, miR-194, miR-215, miR-199-3p and miR-1388a-3p. In fact, out of the 29 mRNAs significantly correlated with PU, 13 were identified as enriched with the gene ontology term ‘regulation of phosphorus metabolic process' (GO: 0051174), including *SMAD3, CAV1, PPP1R1A, ATP2B4 *and* PKD1*.

*ATP2B4* and *PKD1* are involved in intestinal calcium uptake and, thus, are closely related to P homeostasis in terms of a constant cellular Ca : P ratio. *ATP2B4* is located in the plasma membrane and promotes intestinal Ca transport [25]. For *PKD1* knock-out mice, the abundance of this gene was shown to affect the renal expression of several genes involved in mineral homeostasis, including *ATP2B4*, and consequently leads to altered serum mineral levels [26]. *SMAD3* expression was reported as crucial for osteogenesis in studies of stem cell differentiation into cells with an osteoblastic phenotype [27]. The rate of osteogenic differentiation was decreased when *SMAD3* signalling was impaired [27]. In addition, *SMAD3* cis-acting eQTL operating in primary osteoarthritis and in the aneurysms and osteoarthritis syndrome was reported [28]. In addition, *SMAD3* directly mediates TGF-β signalling which is active and regulates the cellular functions of intestinal epithelial cell [29]. Smad3–/– mice showed defective transforming growth factor β-signalling and associated with disease conditions of the gut [30,31]. In our study of Japanese quail, *SMAD3* showed a cis-mRNA-eQTL (*p*-value = 1.5 × 10^−15^) located on chromosome 10 (position 171 kb) with the highest association of SNP_ID18249. The expression of this gene was also significantly correlated with PU, CaU, FI, FCR and BWG. The gene was revealed in our previous study and provides an intestinally expressed candidate molecule for divergent PU in quails [2]. Putative mechanisms are the interaction of SMAD3 with the vitamin D receptor and the initiating of effects mediated by vitamin D3 [32]. Moreover, *SMAD3* showed a significantly negative correlation with miR-191-5p (*r* = −0.23, *p* < 2 × 10^−7^) followed by miR-215-5p (*r* = −0.15, *p* < 7 × 10^−4^).

*ENPP6* and miR-16-5p were significantly correlated with PU, CaU, FI, FCR and BWG. A cis-eQTL of miR-16-5p mapped to chromosome 1 (position 151.6 kb) with two SNPs (id10120 and id07989) (chromosome 1 position 152 kb) being associated at *p* = 2.0 × 10^−4^. A previous study reported that insertion mutation disrupts genesis of miR-16 and causes increased body weight in domesticated chicken [33]. *ENPP6* has also a cis-eQTL located on chromosome 4 (position 35 kb) with SNP id17811. Both miR-16-5p and *ENPP6* were reported in our previous study to link to PU [2]. *ENPP6* activities mediate bone mineralization which is a key process in the formation of bone [34]. In addition, *ENPP6* was found positively correlated with *Candidatus *Arthromitus** while miR-16-5p positively correlated to *Enterobacter*.

The link between the host and gut microbiota can be mediated by miRNA. Due to their strong evolutionary conservation, faecal miRNA-mediated inter-species gene regulation facilitates host control of the gut microbiota [35]. In other case, microbiota-derived metabolites may influence the host's cellular functions. Recently, a study reported that microbiota-derived nitric oxide directly alters the host's Argonaute family protein activity, and consequently altered miRNAs and gene expression of the host [36]. *CAV1* belong to nitric oxide (NO) signalling and negatively regulates NO synthase activity [37,38]. The reduction in NO concentration in the gut of mice after treatment with a polysaccharide from Spirulina platensis was accompanied by an increase in the abundance of beneficial bacteria, including *Candidatus *Arthromitus** [39]. *Candidatus *Arthromitus** belongs to segmented filamentous bacteria (SFB) and selectively attach to the ileal epithelium of the host [40]. SFB genomes contain genes predicted for two catalases, a peroxidase and an arginase, which might limit nitric oxide production through catabolism of arginine [41,42]. The previous study showed the role of SFBs (in particular *Candidatus *Arthromitus**) in the ileum of turkeys that were related to weight gain which is also in line with our study in Japanese quail [43]. The previous study of the same animals demonstrated the impact of the microbiota composition on quantitative trait variation in particular PU, CAU, FI, FCR and BWG [21]. The integration of analyses of microbiota abundances with the genetic variation of the host, transcript levels and complex traits reveals new relationships. For instance, the microbiome-wide association analyses to study the relationship of abundances of microbiota with other traits revealed association of some genera including *Candidatus *Arthromitus** with multiple traits [21]. In our study, *CAV1* ct value was negatively correlated to miR-1388a-5p and miR-1388-3p expression and positively correlated with *Candidatus *Arthromitus** abundance. We found *CAV1*, miR-1388a-5p, miR-1388-3p and *Candidatus *Arthromitus** being linked to the phenotype of BWG, FI, FCR and PU. We also found that the miR-1388a-5p and miR-1388-3p were highly heritable.

The correlations shown here provide further evidence for the complex interactions that exist between microbiota, mRNA and miRNA expression, and phenotypes, which in line with detailed previously described connections. In particular, we found evidence that the interaction of miRNAs and mRNAs and the gut microbiota play an important role in PU and other related phenotypes. In addition, this study demonstrated genetic regulation by eQTL analysis and the heritability of expression of both miRNAs and mRNAs correlated with mineral utilization and other related traits and associated with variable microbiota abundances. The study also revealed that approaches to improve a complex trait such as PU, which allow for resource-efficient and environmentally friendly animal husbandry, need to consider multiple levels of the genotype–phenotype map.

## 4. Materials and methods

### 4.1. Experimental design and samples selection

Japanese quails (*Coturnix japonica*) used in this study were originated from a previous study [18,19]. The experiment was conducted in accordance with the German Animal Welfare Legislation approved by the Animal Welfare Commissioner of the University of Hohenheim. An F2 design using two Japanese quail lines divergently selected on social reinstatement behaviour was established with 12 males from line A and 12 females from line B of the F0 generation. A total of 17 roosters and 34 hens were randomly selected from the F1 birds to generate F2 animals [18]. In order to let the birds express their full genetic potential of PU, the F2 animals were fed with a low-P diet (4.0 g P kg^−1^ dry matter) without a mineral P supplement or phytase. In total, 482 birds of the F2 quail population were used. All the traits used in this study including PU, CaU, were descripted in detail by Beck *et al*. [18]. In brief, PU was calculated as PU [%] = 100–100 × [(PExcreta)/(PDiet × FC)], where PExcreta and PDiet were the analysed quantities of P in the collected faeces [mg] and in the feed [mg g^−1^], respectively, and FC was the individual feed intake measured over a 5-day collection period [g] [18]. The corresponding calculation applies for CaU.

### 4.2. RNA extraction and quantitative PCR

After feeding the low-P diet for a period of 10 days and when the animals were 15 days old, the ileum samples were dissected, cut open and rinsed with a sterilized saline buffer to remove digesta residue. The intestinal epithelium was scraped using sterilized razor blades and were immediately submerged in RNAlater solution (Sigma) and stored at −80°C until RNA extraction. Total RNA was extracted from approximately 50 mg sample using TRIzol Reagent (Invitrogen) and the RNeasy Mini kit (Qiagen) with DNaseI treatment according to manufacturer's recommendations for mRNA. A 2100 Bioanalyzer Instrument (Agilent, Santa Clara, CA) was used for RNA quality assessment. The cDNA synthesis of miRNA was performed according to a previous study [44]. In brief, 100 ng of total RNA were poly(A) tailed and reverse transcribed using 1 unit of poly(A) polymerase 1 µM (BioLab), RT-primers (CAGGTCCAGTTTTTTTTTTTTTTTVN where V is A, C and G and N is A, C, G and T), 0.1 mM of NTPs, 100 units of MuLV reverse transcriptase (Invitrogen). The reaction was incubated at 42°C for 1 h followed by 95°C to inactivate the enzyme. For cDNA synthesis from mRNAs 200 ng mRNA was mixed with 1 µl Reverse Transcription Master Mix (Fluidigm PN 100-6297) in 5 µl volume. The reaction was incubated at 25°C for 5 min, 42°C for 30 min followed by 85°C for 5 min. The cDNA was used for further quantitative PCR (qPCR).

In total, 77 miRNA and 80 mRNA from 482 F2-quail samples were used for qPCR with the Fluidigm BioMark HD System. Specific target amplification (STA) was done per manufacturer's recommendations. Pre-amplification sample mixtures were prepared using PreAmp Master Mix (Fluidigm PN 1005581) containing 1.25 µl of cDNA, 1 µl PreAmp Master Mix, and 0.5 µl Pooled Delta Gene Assay Mix (500 nM) containing DNA suspension buffer and primers mixes (electronic supplementary material, file S1 for miRNA and electronic supplementary material, file S2 for mRNA) in 5 µl total volume. The pre-amplification reaction was incubated at 95°C for 2 min, followed by 10 cycles at 95°C for 15 s and 60°C for 4 min. The pre-amplification reaction was cleaned up using exonuclease I, followed by 10 × dilution of STA with DNA suspension buffer (TEKnova, PN T0221). Fluidigm quantitative measurement runs were carried out with 96.96 dynamic arrays (Fluidigm Corporation, CA, USA) according to manufactures instructions. In brief, 2.5 µl of 2 × SsoFast Evagreen Supermix with Low ROX, 0.25 µl 20 × sample-loading reagent, and 2.25 µl of treated samples were prepared. Separately, an assay mixture was prepared for each primer pair and this included 2.25 µl of DNA Suspension buffer, 0.25 µl of 100 µM forward and reverse primer and 2.5 µl of 2 × assay-loading reagent. The dynamic arrays were first primed with control line fluid and then loaded with the sample and assay mixtures via the appropriate inlets using an IFC controller. The array chips were placed in the BioMark Instrument for PCR at 95°C for 10 min, followed by 30 cycles at 95°C for 15 s and 60°C for 1 min. The data were analysed with real-time PCR analysis software in the BioMark HD instrument (Fluidigm Corporation, San Francisco, CA). The internal controls of cel-miR-39-3p, *18S*, *SNORD21* were used for miRNA and six housekeeping gene (*RAB35, RPS11, ACTB, AGFG1, UB2A* and *UBE2D1*) were used for mRNA.

### 4.3. eQTL detection

Genotyping data were generated in a parallel genome-wide association and linkage study of PU and related traits [45]. In brief, genotyping was performed using costumer's Illumina iSelect Chips for 5388 SNPs genotyped. After normalization and filtering, 3469 SNPs of the 482 individuals were used. Based on the estimated linkage map of these SNPs markers, this map was updated and compared with the new reference genome of the Japanese quail (*Coturnix japonica* 2.0) [45]. The 77 miRNA and 80 mRNA Ct values and SNPs of 482 individuals were subjected to a mixed-model analysis of variance using JMP Genomics (Proc Mixed; SAS Institute, Rockville, MD). An identity by descent (IBD) matrix giving the relationships between individuals was used as a random effect. Additionally, genotype, gender and the batch of RT-PCR were used as fixed effects. Ct values of 18S, SNORD21 and cel-miR-39-3p and the housekeeping genes were used as covariates for analyses of miRNAs and mRNAs, respectively. To correct for multiple testing, a FDR at 10% was used for miRNA and 5% for mRNA. We defined an eQTL as ‘cis' if an associated SNP was located within an area less than 10 Mb from the corresponding gene. All other eQTL were considered as ‘trans'.

### 4.4. Genomic heritability estimation

Genomic heritability represents the proportion of genetic variance explained by SNPs to the phenotypic variant (i.e. miRNA or mRNA expression), and was calculated using the JMP Genomics. The estimated genetic influence on traits was based on the SNP data matrix instead of using a formal experimental design [46]. The measured SNP-level variation is used to estimate the genetic similarity between individuals, and this estimated genetic similarity is compared to the phenotypic similarity to produce a heritability estimate. To estimate the heritability of the expression for a specific miRNA or mRNA, sex and technical covariates were included as a fixed effect, the genetic similarity matrix between individuals was first computed as identity-by-descent of each pair for the k-matrix (SNPs) used as the random effect.

### 4.5. Trait-correlated ileum transcripts expression and gut microbiota

Operational taxonomic units (OTUs) deduced from 16S rRNA sequencing of the same animals was obtained from a recent study [20]. Initially, OTUs were assigned to taxa at the genus level and OTU counts belonging to the same genera were summarized. Moreover, the dataset was filtered so that only taxa with more than one observation in at least half of the samples were considered. In total 91 gut microbiota genera from 411 individuals were used for the correlation study. After adjustment for the effect of either miRNA- or mRNA expression, gut microbiota abundances or phenotypic traits as described above, the residuals were retained for further analysis. The correlation of miRNA or mRNA Ct values as well as gut microbiota with PU and other related traits were estimated as Pearson coefficients and corrected for multiple comparisons by calculating the FDR [47]. Networks of mRNA, miRNA, gut microbiota and the phenotype were visualized with cytoscpe (http://cytoscape.org).

## Data accessibility

All data are available in the electronic supplementary material.
